# Perspectives of Spanish-Speaking Women on Telemedicine for Contraceptive Care

**DOI:** 10.1097/og9.0000000000000065

**Published:** 2025-02-27

**Authors:** Cynthia Coots, Adriana Mesa, Richard Gallardo, José Juan Vicéns-Villafaña, Bianca M. Stifani

**Affiliations:** Westchester Medical Center and New York Medical College, Valhalla, New York.

## Abstract

**OBJECTIVE:**

The use of telemedicine for reproductive health increased during the coronavirus disease 2019 (COVID-19) pandemic, but people with language barriers are less likely than others to use it. The objective of this study is to describe the perspective of Spanish-speaking immigrant women on the use of telemedicine to access reproductive health services, including contraception.

**METHODS:**

We conducted a qualitative study using in-person and virtual focus groups with primarily Spanish-speaking reproductive-aged women who reside in a suburban area outside of New York City. We recruited participants through flyers, social media, and a partnership with a community-based organization. We analyzed interview transcripts using grounded theory methodology.

**RESULTS:**

Between May and November 2022, we conducted six in-person and two virtual focus groups with a total of 58 participants. Most participants were from Mexico and Guatemala and had limited experience with telemedicine. Participants recognized that telemedicine has practical advantages, including economic ones. However, many expressed concerns about the quality of virtual health care, which they felt led to “guesswork,” because it is not possible to conduct a physical examination. When prompted, participants recognized that telemedicine could be a viable option to provide information about different contraceptive options and to initiate some contraceptive methods such as pills. Low technological literacy and lack of access to cell service were often cited as barriers to using telemedicine.

**CONCLUSION:**

To ensure equal access to telemedicine among underserved populations, it is important to understand their needs and perspectives. Educating prospective users about the value and limitations of telemedicine and overcoming technological barriers are essential steps in ensuring digital health equity.

## Abstract 2

Spanish-speaking immigrant women see the potential of telemedicine for contraceptive care but are concerned about the quality of care and cite multiple barriers to access.

During the coronavirus disease 2019 (COVID-19) pandemic, clinicians embraced telemedicine to decrease viral exposure for themselves and their patients.^1^ In spring 2020, family planning specialists across the country, most of whom had never used telemedicine before, began providing contraceptive counseling and other related services using remote technology.^2^ Although telemedicine previously had been used to provide abortion care,^3,4^ its use for contraceptive care was new. Initial findings describing patient experiences with virtual contraceptive care were encouraging.^5^ Clinicians also were enthusiastic about this new service, but they expressed concern about patients who may be unable to use it due to limited access to the required technology. Some also worried about challenges specific to patients with low English proficiency.^2^

Studies in family planning and other fields have since demonstrated that the utilization of telemedicine is lower in certain demographic groups.^6^ One analysis of telemedicine visits in primary care found that older patients and patients with low English proficiency were significantly less likely to use telemedicine and that Black, Latinx, and poorer patients had less video use.^7^ A study of pediatric visits reported similar findings, with Black and Latinx patients and patients with low English proficiency having fewer telemedicine visits and patients with public insurance being less likely to access video visits.^8^ One study that examined access to telemedicine for contraceptive care in California found lower utilization among people who speak Spanish.^9^ A nationwide social media survey of reproductive health described lower utilization and poorer quality of virtual contraceptive care among Hispanic and Latinx individuals.^10^

These findings point to troubling inequities in access to health care using telemedicine. Yet little is known about what drives these inequities, and about how they can be overcome. Crawford and Serhal^11^ have proposed a digital health equity framework outlining digital determinants of health to be addressed to achieve digital health equity. These determinants are, “access to digital resources,” “use of digital resources for health seeking or health avoidance,” “digital health literacy,” “beliefs about potential for digital health to be helpful or harmful,” “values and cultural norms/preferences for use of digital resources,” and “integration of digital resources into community and health infrastructure.”^11^ To understand inequities in telemedicine usage for reproductive health care, we designed a study exploring the digital determinants of health among Spanish-speaking immigrant women in New York.

## METHODS

We conducted a focus-group based, qualitative study to explore the perspectives of Spanish-speaking women on the use of telemedicine for reproductive health services including contraception. We report our methods and findings according to the guidelines set forth by the COREQ (Consolidated Criteria for Reporting Qualitative Research) checklist.^12^

The setting for this study was Westchester County, New York, which is located at the northern border of New York City. Approximately 1 million people live in the county, which has urban, suburban, and rural areas.^13^ Public transportation options are limited. The Hispanic population has been growing in this region. As of 2022, one-quarter of the county's population identified as Hispanic, and a quarter of these individuals do not speak English well.^14^ The most common places of origin for those who are foreign-born are Mexico, the Dominican Republic, and Ecuador.^15^ Although Westchester County is home to some of the wealthiest neighborhoods in the nation, most of its Hispanic residents live in towns where the median household income is well below the national average.^16^

For this study, we recruited reproductive-aged women (age 18–45 years) who were primarily Spanish-speaking and who resided in Westchester County, New York. Recruitment materials used the word “woman,” without specification with regard to “cis-” or “trans.” We recruited participants from community and clinical settings. We partnered with a community-based organization based in Mamaroneck, whose staff invited individual organization members to participate in the study. This organization helps immigrant families and families with low income become self-reliant by providing them with resources, education, and advocacy. We posted flyers at clinical sites where the investigators work and at community-based clinics, as well as grocery stores, nail salons, and community events. We also posted advertisements on local social media groups. Interested women contacted the study team by phone, email, or text to obtain more information, complete screening for eligibility, and schedule participation in an upcoming focus group. We offered participants a $50 Visa gift card as a token of appreciation for their time.

We conducted in-person and virtual focus groups with 6 to 10 participants per group. We designed a focus group facilitator guide based on our prior experience conducting focus groups and research about telemedicine for reproductive health. The guide asked about experiences accessing reproductive health care in the United States and about experiences with telemedicine. This included questions relating to participant experiences with language barriers when accessing both in-person and telehealth care. We asked participants specifically about telemedicine for contraceptive care. The guide also included two graphics that showed different modalities for scheduling and accessing telemedicine visits, which we used to elicit participants' preferences. The study team designed all study materials in Spanish and translated them into English for the purposes of ethics approval. A certified professional translator verified all translations. The New York Medical College Institutional Review Board approved this study, which was funded by the Society of Family Planning Research Fund.

Trained facilitators (B.M.S., A.M., J.J.V.-V., C.C.) who are fluent in Spanish conducted the focus groups. B.M.S. is an obstetrician–gynecologist and complex family planning specialist with extensive training and experience in qualitative research. C.C. is an obstetrics and gynecology resident, A.M. is a medical student, and J.J.V.-V. is a practicing obstetrician–gynecologist. All received training on focus group facilitation during this project. None of the facilitators had preexisting relationships with any focus group participants. We conducted in-person focus groups in a space provided by our community partner, and we hosted virtual focus groups using Zoom. Each focus group lasted approximately 2 hours. We collected demographic information for each focus group participant. We recorded and transcribed each focus group.

We analyzed the data for this study according to the principles of grounded theory, a systematic, inductive, and comparative approach of inquiry for the purpose of constructing theory.^17,18^ In grounded theory, data collection and analysis proceed simultaneously, as researchers move back and forth between empirical data and emerging analysis.^18,19^ We began the data-analysis process with open coding, letting codes emerge from the data rather than using preconceived codes.^17–19^ The entire data-analysis team (B.M.S., C.C., A.M., R.G.) coded the first two transcripts and agreed on a codebook, which we then applied to the remainder of the transcripts. Two researchers coded each transcript. We then grouped and organized codes into a higher level of abstraction and agreed on key themes that emerged from the data. Throughout the process, we engaged in memo writing and held research meetings to reflect on our positionality and how it affected our reading of the data.^18^ We began coding after the third focus group was completed and monitored our data for the achievement of thematic saturation. When the entire research team agreed that no new themes were emerging, we stopped recruitment. We conducted all data analysis in Spanish and translated participant quotes for this article. We used Atlas TI Cloud for the data-analysis process.

## RESULTS

We conducted eight focus groups (six in-person, two virtual) with a total of 58 participants (43 in-person, 15 virtual) from July to November of 2022. We recruited most participants through our partnership with the community-based organization. Many were domestic workers, and the more common countries of origin were Guatemala (36.2%) and Mexico (34.5%). See Table 1 for participant demographic characteristics.

**Table 1.** Demographic Characteristics of the Women Who Participated in Focus Groups About the Role of Telemedicine in Reproductive Health (N=58)

Characteristic	Value
Age (mean, SD)	36±8
Country of origin	
Guatemala	21 (36.2)
Mexico	20 (34.5)
Ecuador	6 (10.3)
Other	11 (19.0)
Time in United States (y)	
Less than 5	10 (17.2)
5–10	11 (19.0)
More than 10	37 (63.8)
Education	
None	3 (5.2)
Elementary	24 (41.4)
Middle school or high school	21 (36.2)
University	8 (13.8)
Other	2 (3.4)
Occupation	
Housekeeper	21 (36.2)
Child or eldercare	7 (12.1)
Stay-at-home parent	12 (20.7)
Unemployed	5 (8.6)
Other	12 (20.7)
Access to transportation	
Yes	21 (36.2)
No	33 (56.9)
Sometimes	4 (6.9)
Access to smartphone data	
Yes	47 (81.0)
No	3 (5.2)
Sometimes	8 (13.8)

Data are mean±SD or n (%).

During focus group discussions, many participants did not recognize the term “telemedicine” and reported no or limited experience with video or telephone visits. The participants who reported such experiences stated that during the COVID-19 pandemic virtual visits were the “only option” because clinics were not offering in-person visits. None were given the option of an in-person or virtual visit. Most telemedicine experiences reported were in pediatrics or general medicine (well-child or sick visits often related to COVID-19 symptoms). A few patients had received some virtual prenatal care, but none had used telemedicine to access other sexual or reproductive health services (including contraception). Many of the participants routinely participated in community meetings through Zoom and were, therefore, familiar with the video conferencing platform.

Focus group participants, even those who had not experienced telemedicine, generally agreed that there are practical advantages to virtual care. Many pointed to lack of transportation and difficulties navigating public transit as barriers to health care in their communities, especially for those recently arrived from other countries (“When you get here, you start from zero, it's hard to figure out where to take the bus, where the train goes…”). In this geography, participants described limited public transportation options (“There are buses, but they don't exactly get to the hospitals, and they don't run at night”) and unreliable schedules (“The bus comes once an hour”), leading to late arrivals and missed appointments (“I either get there 1 hour early and I have to wait, or I get there half an hour late and they cancel my appointment.”). With telemedicine, these challenges can be avoided:

“[Telemedicine saves time] because, imagine, in my case, to go to an appointment, I have to take a bus that takes 45 minutes, then wait 45 minutes [for the appointment], then wait another 45 minutes for the bus back. And it's 3 hours before I'm back home.”

Relying on family members for transportation was common. As one participant said:

“In my case, if I have to go somewhere, my husband has to ask for a day off from work and then there's no one to take care of the children, so the whole family has to go to mom's doctor's appointment.”

Participants also explained that telemedicine is economically advantageous, because taking time off work for appointments can be avoided:

“[Telemedicine] is favorable for many people because [for example] they can have their visits during their lunch break and not have to ask for an entire day off—because if you take a whole day, you don't earn anything for that day.”

Virtual care allows people, “to not lose the whole day [because they] can do it any time, in the comfort of [their] home, in the street, or wherever [they] are, it's fast.” For those who have children, not having to seek childcare can also be advantageous:

“I have three kids, my youngest is 6 months [and during my pregnancy] it was very difficult to get transportation, so it was very good to have a medical check-up via videocall. Then, when I had to have something physical like an ultrasound, I liked to go in person, but like in my case—a pregnant woman who has other children—I think [telemedicine] was very good.”

Some of the participants who had tried telemedicine also felt that these appointments could be scheduled more quickly than in-person appointments and had fewer unexpected delays (“You just wait for the call, and you get connected.” and “You don't have to be in there waiting for them to see you.”).

Despite telemedicine's practical advantages, many participants expressed concern about the quality of care provided virtually. A few participants recounted personal or family members' experiences with telemedicine consultations resulting in wrong diagnoses or otherwise poor care. Some felt that their concerns and symptoms were dismissed only to result in serious illnesses down the road. Others didn't experience specific negative consequences but did not feel adequately cared for. For example, one woman said:

“When I was pregnant, due to the times [COVID-19 pandemic] we couldn't go out, so that's how they saw me [on the screen]. They would just ask me how I felt, how I was, and they would tell me what I had to do. I wanted them to examine me because I felt my pregnancy was complicated, but they would only see me via phone, and I don't feel like they took good care of me.”

Many participants felt that they had often received poor care in the United States because of their race, their lack of insurance, and the fact that they did not speak English. These experiences were not specific to telemedicine. But the concern that was specific to virtual visits was about the lack of physical examinations leading to poor quality care. As several participants explained:

“I think in-person care is better because they are looking at you and examining you. But virtual, like that, no—because they are only seeing you on the screen and not examining you. I saw that my son was sick, he had a rash—but they only saw him on the phone, and they didn't put the apparatus on him to examine him. They just gave him a medicine and it didn't work.”

“I hope telemedicine no longer exists in the future, because it’s guesswork…internet, phone…it's not safe! Patients need to be examined, at least a blood pressure measurement!”

“I don't think telemedicine can be used for sexual and reproductive health, because I feel that it is something very intimate between the patient and the doctor…I feel that the doctor should feel or see, check in-person to be able to give the results with a higher percentage of effectiveness, or to not make a mistake. Because I believe that, through a screen, sometimes things are distorted, one looks strange, and there may be errors and perhaps bad results.”

Several participants felt that patients need “to be checked” physically, even when they are not sick. This came up when we specifically asked them to consider telemedicine as an option for contraceptive care. One participant recalled that she previously had been required to come into a clinic for a physical examination before receiving a prescription for contraception:

“I was told [that you always need a check-up] before getting birth control. I went to the [clinic] and told them ‘I need the pills,’ and they said, ‘we’ll make you an appointment so you come for your checkup first; we can't just give you pills like that’…They also have to make sure you're not pregnant because, if they give you something and you are, it's not good.”

Given this experience, the participant believed that a physical check-up is always required for initiating contraception. In general, focus group participants seemed to value the physical examination not only during sick visits but also as an opportunity to detect previously unknown health issues. Consensus was high among group participants about the lack of physical examinations being a significant limitation of telemedicine. However, only a handful recounted lived experiences that corroborated this fear and fewer still had personally experienced this issue as a patient. For most, this remained a theoretical concern.

Technological barriers to accessing telemedicine came up frequently in focus group discussions. The most common issues were lack of familiarity with the technology and lack of access to cell phone service or internet data (either because of financial constraints or poor service in certain geographies). Several participants explained that they had first learned about videoconferencing technologies through their children's schools operating remotely at the height of the COVID-19 pandemic. Some did not own tablets or laptop computers but received them through their children's schools. Asking the children for help in using the devices and navigating the technology was not uncommon. As one woman said:

“I have also had difficulties because I don't understand the technology these days. Only when my son arrives and I ask his help, he tells me how I should access some page, or what I have to click to enter [the televisit].”

When asked about different modalities for accessing telemedicine visits, several participants expressed concerns about learning how to use an unfamiliar technology (“I'm worried I would not understand how to connect to these visits via phone, video, computer…”). Some were concerned about being unable to access the visit at the scheduled time and missing the appointment. Several preferred a telemedicine visit modality in which they would simply receive a phone call because this wouldn't require learning how to navigate the technology (“I would prefer a phone call. Because I am very bad with technology. I just wouldn't understand”). Several participants recognized their current limitations but stated they wanted to improve their technological literacy (“If this technology is coming, we have to learn”).

Focus group participants also encountered significant barriers due to intermittent or no access to cell phone data. As two participants explained:

“I run out of [cellular] data, and [my phone] doesn't work anymore. Then I have to look for a wi-fi somewhere else or wait until I get home to be able to send messages. Or I have to see them on the children's electronic devices.”

“I think that most of the time the problem to enter the video calls or video conferences is the internet problem. Most of the time we don't pay on time for the internet, or many things, there are many reasons. Because that's when suddenly you have an emergency and there is no internet. That has happened to me.”

Not being able to afford cell phone plans was a common problem; when mentioned, it was met with high levels of agreement among group participants. Some also felt that this was a prevalent issue throughout their community (“I think most of us have this problem of lack of data and memory in our phone, because you have to pay for storage on your phone”).

But economics were not the only barrier to cell phone service. Several participants also said they lived or worked in areas with limited cellular data coverage and did not have access to other alternatives:

“In my house there is no reception; sometimes it comes, but then it goes. I learned that when I have a visit with a doctor via phone, I have to go out of the house or else they'll hang up after saying, ‘I can't hear you! I can't hear you!’…so I go out to the street, just like in our [home] countries. [And when it's cold] I sit in the car.”

“I don't have a good data network in my cell phone or at home, so it is difficult for me in that way, so I don't like telemedicine; I prefer everything in person.”

For some, connectivity issues resulted in missed appointments. One participant recounted spending half an hour trying to connect to a nutritionist appointment, not understanding whether the issue was on her end or the health care professional's; she ultimately realized her wi-fi was not working and had to reschedule the appointment. Another said that sometimes simply because it's raining, “The signal goes away, and there goes the visit.”

None of our focus group participants had experienced telemedicine specifically for accessing contraceptive care. However, when asked to imagine whether this service could work, several recognized that virtual visits could be used as an option to learn about available contraceptive options. Despite many participants having expressed concern about telemedicine for reproductive health care in general (as described above), consensus was high in most groups when the discussion shifted to contraceptive provision more specifically. Participants recognized that much of contraceptive care is counseling and education, which can be effectively accomplished virtually:

“I don't think [anything] gets lost because I'm going to pay attention to [the health care professional] in the same way if it's via phone or video, and I can learn about contraception. For example, I never knew there was an implant [to prevent pregnancy], and I learned that not too long ago.”

“[Telemedicine] is a good idea for when you're just getting started…because, even us adults, we don't know about certain [contraceptive] methods. I think it would be a good visit if they first explain [the method] and then they give it, because that way you can choose and decide what's best for you and what's least likely to harm you.”

Participants also saw telemedicine as appropriate for hearing about the potential side effects of different methods:

“It seems to me that, when it comes to family planning methods, this telemedicine method would be very good because I can tell the doctor this method makes me fat, this method hurts me, I get more time, less time; in other words, there is no need to go to the doctor's office.”

“When I had my last child, I was very interested in what birth control method I was going to use, and it was a problem because they just gave me a sheet and told me to read it and pick which one I wanted. I think it's important to know how one's body is because for example I have irregular periods…so what works for me may not work for another woman…So to have a videocall where someone asks me about my cycle, my weight, my height, my hormonal symptoms and all that and tells me, ‘this is what I recommend for you, it will not hurt your self-esteem or your weight…’ A visit like that would be very good.”

One participant thought that, if the counseling was done virtually, it could shorten the in-person visit time if one was required:

“It’s like speeding up the process. The first consult by telephone, and then you're done. Because, if we make the decision to use an IUD…they already gave you the explanation, the advantages, the disadvantages, you decided to use it, they tell you, ‘come Thursday at 8 am.’ You don't go for just one part. It would save a lot of time.”

Several participants also recognized that telemedicine was an appropriate way to obtain some but not all contraceptive methods:

“Telemedicine is ok for pills, condoms, injections in case someone can give it. I think that would be the only thing. Other than that, I think I would physically need to be present.”

“If you're getting pills, you don't need to go in. You can save your time and the doctor's time.”

## DISCUSSION

In this qualitative study, we found that Spanish-speaking focus group participants with limited telemedicine experience recognized the practical advantages of virtual care but were concerned about the quality of care provided in the absence of physical examinations. Participants described barriers to accessing the technology but recognized its potential for learning about contraception and obtaining some contraceptive methods. It is an encouraging finding that many of our participants were able to envision a role for telemedicine in the provision of contraceptive education and counseling and were open to trying it themselves.

A great facilitator to adopting virtual care has to do with its practical advantages. Our participants' recognition of these benefits resonates with prior findings from the literature. Telemedicine has been extensively studied as a means of improving access to care because it eliminates requirements for transportation and other logistical aspects of care-seeking. It has been successful at reducing geographical barriers to accessing health care.^20^ Across specialties, telemedicine has been shown to be cost effective and to maintain or even improve the standard of care and associated health outcomes.^21,22^ This has been achieved while reducing patients' time away from work or school and travel costs and increasing patient satisfaction.^23,24^

On the other hand, our focus group participants' concern about the poor quality of virtual care was an interesting finding that is not in line with prior research in many fields of medicine, which demonstrated high patient satisfaction rates and good patient outcomes with telemedicine. A systematic review of randomized controlled trials comparing telemedicine with in-person care in pediatrics, for example, described comparable or better outcomes with telemedicine for various health conditions.^25^ When it comes to contraceptive care, at least two studies have assessed the quality of contraceptive counseling using the IQFP (Interpersonal Quality of Family Planning) scale for people selecting in-person visits compared with telephone or video visits. Both found similarly high IQFP scores for telemedicine and in-person visits.^26,27^ In our study, most participants had not accessed reproductive health care using telemedicine, and their concerns were theoretical. It is possible that participants’ prior negative experiences with obtaining in-person care in the United States (feeling discriminated against and dismissed in the process, which were common concerns) colored their views of what virtual care experiences may be like. That said, the high value placed on the physical examination is an important finding that is specific to telemedicine. One of the digital determinants of health is “beliefs about the potential for digital health to be helpful or harmful.”^11^ Here, we saw many participants worry that digital health cannot help and can even be harmful because of the lack of physical examination. Implementation of a successful telemedicine program in this population may require providing education about the clinical scenarios in which a physical examination is required or helpful, and those in which it is not.

Other digital determinants of health highlighted by our study were digital health literacy and integration of digital resources into community and health infrastructure.^11^ Our participants had real barriers to accessing virtual care, which were due to both internal (lack of familiarity with the technology) and external (lack of data, lack of cellphone service) factors. This helps explain why Spanish speakers, individuals with low income, and individuals without insurance may have had lower uptake of telemedicine in previous studies.^6–10^ Access to technology is multifaceted, requiring both access to a device such as a computer or smartphone as well as access to internet or cellular service. Data from the Pew Research Center demonstrate that, in 2021, almost all (95%) U.S. adults used the internet. However, although 92% of individuals with household income above $75,000 had access to home broadband, only 58% of those with income below $30,000 did.^28^ Mobile internet access requires cell phone coverage, which is notoriously worse in low-income areas.^29^ Ultimately, decreasing the digital health gap will require a real investment in digital infrastructure.

This study has several limitations. First, most of the participants did not have prior experience with telemedicine for reproductive health, thereby limiting their responses to specific questions to the theoretical realm. Such prior experience was not a requirement for participation or a screening tool for organizing groups with varied perspectives, and the fact that none of our participants had it should be considered an important finding in and of itself. Second, this study is subject to the issues of credibility and transferability that are typical of any qualitative study. Our subjectivity may have affected how we collected data and interpreted interview findings. Our interviewers' position as community outsiders may have affected participant responses, but it was not feasible to recruit community members to lead the focus groups. We took several steps to increase the trustworthiness of our findings, including reflecting on our positionality throughout the research process and double coding all the interviews, but it is impossible to present qualitative data in a completely unbiased manner.

This study also has unique strengths. Our partnership with a local organization to recruit from the community, rather than the clinic, allowed us to engage a population that is not traditionally involved in research. We also were able to hear from individuals who are not already accessing virtual care, which is optimal for understanding the barriers faced by this community. We also conducted the study entirely in Spanish with a Spanish-speaking research team, thus minimizing the effects of language barriers and inaccurate translations on the analysis and interpretation of the findings.

In conclusion, it is encouraging that individuals of low socio-economic status who experience language barriers recognize the potential of telemedicine for contraceptive care, despite having had very limited exposure to this service. Future studies should examine whether the theoretical concerns about telemedicine raised by our participants are also experienced by individuals with similar demographic characteristics once they do access this service. However, this would first require a significant investment addressing the various determinants of digital health to build an accessible and appropriate clinical service. That 81% of participants reported they have access to smartphone data is a promising finding that informs feasibility for telehealth programs for this population, in spite of other barriers. Patient education and improvements in digital infrastructure will be key to achieving digital health equity in reproductive health.
